# Efficacy and Safety of Enoxaparin for Preventing Venous Thromboembolic Events following Urologic Laparoscopic Surgery

**DOI:** 10.1155/2013/415918

**Published:** 2013-06-02

**Authors:** Takeo Nomura, Mika Takahashi, Kazunori Iwasaki, Tomoya Oribe, Mayuka Shinohara, Yuko Fukuda, Shinsuke Mizoguchi, Fuminori Sato, Hiromitsu Mimata

**Affiliations:** Department of Urology, Oita University Faculty of Medicine, 1-1 Idaigaoka, Hasama-machi, Yufu, Oita 879-5593, Japan

## Abstract

There is a paucity of definitive evidence that supports the use of enoxaparin to prevent venous thromboembolism (VTE) after urologic laparoscopic surgery. The purpose of this study was to evaluate the efficacy and safety of postoperative subcutaneous enoxaparin injection in patients who underwent urologic laparoscopic surgery. A total of 63 patients were evaluated from June 2010 to December 2012. All patients received postoperative prophylaxis with enoxaparin (2000 IU twice daily for 5 days). None of the patients treated with enoxaparin developed symptomatic VTE, but two cases (3.2%) of pulmonary embolism were noted before initial enoxaparin administration. Statistically significant differences were observed between the prothrombin time (PT) and activated partial thromboplastin time (APTT) values and D-dimer levels obtained at baseline and on day 7 after surgery; however, the PT and APTT values did not exceed the normal range. In addition, signs of any adverse events were not encountered in any of the patients treated with enoxaparin. The use of enoxaparin immediately after a surgery may confer valuable thromboprophylaxis benefits for urologic laparoscopic surgery.

## 1. Introduction 

Venous thromboembolism (VTE), including pulmonary embolism (PE) and deep venous thrombosis (DVT), is a major complication in patients who have undergone surgery [1]. Although these events are rare, they can be still associated with high mortality during the early postoperative period. Several known significant risk factors are responsible for the development of VTE, including female gender, advanced age, advanced-stage cancer, prolonged surgical duration, intrapelvic surgeries, varicose veins, immobilization, obesity, history of VTE, and a high number of chronic medical comorbidities [2–4]. In particular, patients undergoing curative abdominal cancer surgery are considered to be at a high risk for VTE [5]. In recent years, numerous urologic surgical procedures have been laparoscopically performed, and these offer some advantages over conventional openincisional surgery, including decreased pain, quicker convalescence, and improved cosmesis. Nevertheless, this technique is still associated with a distinct morbidity. Moreover, the incidence of VTE associated with laparoscopic and open incisional surgery has been reported as almost equal [6, 7], but the abdominal insufflation used during laparoscopic procedures has been proposed to cause serum hypercoagulability of varying degrees and VTE secondary to venous stasis [8, 9]. In addition, the patient's position such as the lateral flank position during kidney/adrenal gland surgery or the lithotomy position during prostate/urinary bladder surgery may be another risk factor that predisposes to decreased venous return, thereby increasing the risk of VTE.

The incidence of VTE following a major abdominal surgery without prophylaxis has been reported to be approximately 20%, and the reported incidence of symptomatic PE ranges from 0.5% to 1.6% [4, 10, 11]. Because VTE may rapidly lead to fatality, its prevention by early ambulation, intermittent pneumatic compression (IPC), or chemoprophylaxis is inevitable, particularly in patients with potential risk factors. However, there are no uniform guidelines for the use of chemoprophylaxis, and little evidence is available to justify a routine prophylactic anticoagulation treatment for laparoscopic surgery. Till date, to the best of our knowledge, there have been no randomized controlled trials addressing the issue of VTE prophylaxis in patients undergoing urologic laparoscopic surgery. 

 In the present study, we have evaluated the validity of chemoprophylaxis with subcutaneous administration of enoxaparin for the prevention of VTE in urologic laparoscopic surgery. This is the first report to evaluate detailed laboratory data changes in patients treated with enoxaparin after urologic laparoscopic surgery and is considered to be clinically informative.

## 2. Methods

### 2.1. Patients

This study was performed with the approval of the local institutional review board. Prior to treatment, we obtained verbal and written informed consent from all patients. We evaluated 63 consecutive patients (46 males and 17 females; age, 25–85 years (mean, 66 years); body mass index, 17–34 kg/m^2^ (mean, 23.5 kg/m^2^)) undergoing urologic laparoscopic surgery between June 2010 and December 2012 (Table 1). Of all the laparoscopic surgeries performed, 24 were nephrectomies, 10 adrenalectomies, 9 nephroureterectomies, 5 cystectomies, 5 partial nephrectomies, 4 donor nephrectomies, 2 prostatectomies, 2 nephroureterectomies with cystectomies, 1 pyeloplasty, and 1 renal cyst decortication (Table 2).

Postoperative thromboprophylaxis with a subcutaneous injection of enoxaparin (low molecular weight heparin, LMWH; 2000 IU twice daily) and IPC was planned. Enoxaparin treatment was initiated more than 2 h after the removal of the epidural catheter at 24–36 h after surgery and continued for 5 consecutive days. IPC treatment using pneumatic compressive stockings was initiated on the day of the surgery and continued until patients were completely mobile. All patients were aggressively hydrated and were advised to ambulate within 24 h after surgery. Physical examinations for early detection of VTE and adverse events associated with hemorrhagic complications were performed whenever possible. In addition, hematological examinations (prothrombin time, PT; activated partial thromboplastin time, APTT; and D-dimer levels) were conducted before surgery and on day 7.

### 2.2. Statistical Analysis

Statistical analysis was performed using the Statiew-J4.02 software (Abacus Concepts, Berkley, CA, USA). Unpaired *t*-test was used to evaluate each parameter. The limit for statistical significance was set at *P* < 0.05.

## 3. Results

Table 1 summarizes the patient data. All laparoscopic surgeries were successful. The mean operative time was 312 min, and the estimated blood loss was 251 mL. According to the 8th American College of Chest Physicians (ACCP) Conference on Antithrombotic and Thrombolytic Therapy risk-group classification [12], 1, 6, 40, and 16 patients were classified into low-, intermediate-, high-, and highest-risk groups, respectively. Sixteen patients (25.4%) had concurrent disease such as diabetes, hypertension, heart and respiratory failure, and cerebral infarction. Five patients (7.9%) had a history of abdominal surgery, and none of the patients had prior VTE.

**Figure 1:**
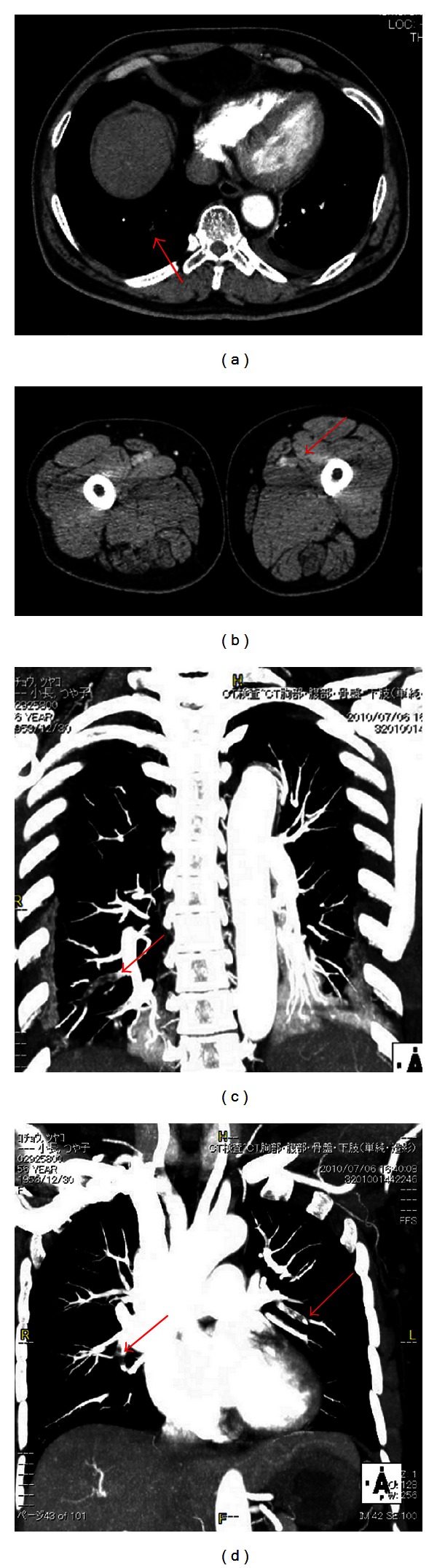
(a) Contrast-enhanced computed tomography revealed the presence of pulmonary embolism (arrow). (b) Contrast-enhanced computed tomography revealed the presence of deep venous thrombosis in left femoral vein (arrow). (c), (d). Contrast-enhanced computed tomography revealed the presence of multiple pulmonary embolisms (arrows).

All patients were administered with postoperative prophylaxis using enoxaparin (2000 IU twice daily for 5 days). Patients treated with enoxaparin did not develop symptomatic VTE, but two cases (3.2%) of PE were noted before the initial enoxaparin administration in this series. A 65-year-old male with left renal cell carcinoma (cT1bN0M0), who had a history of non-insulin-dependent diabetes mellitus, presented with acute dyspnea and decreased oxygen saturation on day 1 after laparoscopic nephrectomy. After a PE originating from the DVT in the left femoral vein was diagnosed, a retrievable inferior vena cava (IVC) filter was placed with the use of anticoagulation (Figures 1(a) and 1(b)). A 56-year-old female with a left renal pelvic urothelial tumor (cT2N0M0) presented with acute shortness of breath and decreased oxygen saturation on day 1 after laparoscopic nephroureterectomy. PE was diagnosed expeditiously using contrast-enhanced chest computed tomography (Figures 1(c) and 1(d)). Immediate anticoagulation treatment with heparin and then with warfarin was initiated. Both patients had an uneventful recovery after anticoagulation therapy.

Our data from hematological examinations performed on day 7 demonstrated a significant decrease in PT (*P* < 0.0001) and APTT (*P* = 0.0108) values and a significant rise in D-dimer levels (*P* < 0.0001) compared with the data before surgery (Table 3). These values did not exceed the normal range or were only marginally elevated and were not considered to pose problems in clinical practice. None of the patients had major bleeding complications or prolonged minor bleeding in this series.

## 4. Discussion

VTE, with the potential sequela of PE, has been recognized as a fatal complication associated with any major abdominal surgery, and the reported incidence of DVT ranges from 15% to 29% for general or gynecologic surgery in the absence of DVT prophylaxis [4, 10, 11]. PE is one of the most common causes of nonsurgical death in patients following surgery, and the frequency of PE has been reported to be between 0.5% and 1.6% [10, 11]. Although there is a paucity of randomized control trials (RCTs) that address this issue in urologic surgery, all patients undergoing urologic surgery have the potential to develop DVT and subsequently PE. The DVT risk in urologic patients undergoing an open pelvic surgery, including radical cystectomy and prostatectomy, was estimated to be 22%–32% without prophylaxis [13, 14], suggesting that these results are similar to the rates of thromboembolic complications associated with other general surgeries. 

In recent years, numerous urologic surgical procedures have been laparoscopically or robotically performed. These procedures offer distinct advantages over conventional open surgery, including decreased pain, quicker convalescence, shorter hospital stay, better cosmesis, and a comparable therapeutic efficacy and acceptable efficiency. Unfortunately, there are no randomized prospective studies that address the development of DVT in urologic laparoscopic surgery; however, several reports in the literature have retrospectively reviewed symptomatic DVT and PE occurrences in patients with prostate cancer undergoing laparoscopic or robot-assisted laparoscopic radical prostatectomy. In one study, there were 31 patients (0.5%) who developed symptomatic DVT including 9 patients (0.2%) with PE among 5951 patients and two patients died of PE [7]. Another study involving a retrospective analysis of patients undergoing laparoscopic or robot-assisted laparoscopic radical prostatectomy found only 2 cases (0.3%) of DVT among 680 patients [15]. In 482 laparoscopic nephrectomies conducted, one PE case (0.2%) was noted, although it is unclear whether any DVT prophylaxis treatments were included [6]. These reports suggest that the DVT risk in urologic laparoscopic surgery appears to be lower, but accurate DVT rates may be higher if screening imaging techniques are utilized rather than clinical observations. 

 Although increasing accumulating evidence demonstrates that DVT does not occur more often with laparoscopic surgery than with open procedures, the abdominal insufflation used during laparoscopic procedures has been proposed to cause serum hypercoagulability of varying degrees and VTE secondary to venous stasis with a concomitant higher risk of DVT and PE [8, 9]. In addition, the patient's position such as the lateral flank position during kidney and adrenal surgeries and the lithotomy position during prostate and urinary bladder surgeries may be another risk factor that predisposes to decreased venous return and increased VTE risk. DVT complications are associated with long-term suffering and postthrombotic syndromes that include pain, heaviness, swelling, varicose veins, leg ulcers, and significant comorbidity, long-term medication, and death in some cases. Although the rates of such complications are low, DVT prophylaxis should be attempted by all conceivable means in all patients undergoing urologic laparoscopic surgical procedures whenever possible.

 In this study, both enoxaparin (2000 IU twice daily for 5 days) and IPC treatment using pneumatic compressive stockings were administered in all patients. In general, therapeutic measures for thromboprophylaxis provide two options, nonpharmacologic physiotherapy that includes early ambulation, graduated compression stockings, and IPS or pharmacologic agents that include low-dose unfractionated heparin (LDUH) and LMWH. Considerable controversy exists regarding the significance of pharmacologic prevention against VTE during laparoscopic surgery because of the low VTE incidence, risk of hemorrhagic complications associated with such agents, and the cost-effectiveness of prophylaxis. The American Urological Association (AUA) recommends the use of IPC devices before laparoscopic surgery or robotically assisted urologic procedures in all patients. In addition, noting the lack of large RCTs, high-risk groups may require the use of LDUH or LMWH before, during, or after surgical procedures [16]. In contrast, Van Hemelrijck et al. concluded that both physiotherapeutic and pharmacological prophylaxis should be used after all major surgeries including laparoscopic surgery for prostate cancer [17]. Furthermore, in guidelines published by the Society of American Gastrointestinal and Endoscopic Surgeons (SAGES), the use of LMWH is recommended as an option for all types of laparoscopic surgery [18]. In addition, a recent report on efficacy of enoxaparin in patients undergoing abdominal or pelvic cancer surgery has indicated that enoxaparin can offer patients an advantage over using IPC alone for VTE prevention [19].

 The use of pharmacologic agents may increase the incidence of hemorrhagic complications during surgery. Moreover, the occurrence of spinal epidural hematoma when using enoxaparin with epidural or spinal anesthesia was reported [20], but enoxaparin has a reduced risk of heparin-induced thrombocytopenia and hemorrhagic complications, severe bleeding, or wound hematomas compared with LDUH in large RCTs [21, 22]. Because our data from hematological examinations and clinical observations indicate that coagulability was not excessively affected by enoxaparin, we propose that this treatment is safe and efficacious without the need for laboratory monitoring of patients when appropriately used. Unfortunately, symptomatic PE occurred before the initial enoxaparin administration in two patients exhibiting additional risk factors such as malignancy and longer surgical duration (350 and 370 min, resp.) in this study. Although our results do not support enoxaparin administration for all patients undergoing a urologic laparoscopic surgery, an initial enoxaparin administration before surgery or immediately after surgery might be considered for high-risk patients. 

## 5. Conclusions

In the present study, we evaluated the validity of chemoprophylaxis using enoxaparin for the prevention of VTE in patients undergoing urologic laparoscopic surgery. With the exception of economic limitations, this approach might be a valuable tool for prevention of perioperative thromboembolic complications. To select the most adequate type of DVT prophylaxis in patients undergoing urologic laparoscopic surgery, the establishment of an appropriate prophylactic regimen and patient risk stratification is required. 

## Figures and Tables

**Table 1:** Patient data.

	Mean ± SD	Range
Mean age (years)	66 ± 13	25–85
Gender (male/female)	46/17	
Body mass index (kg/m^2^)	23.5 ± 3.3	17–34
Operative time (min)	312 ± 144	89–727
Estimated blood loss (mL)	251 ± 365	10–1690
Concurrent disease (*n*)	16 (25.4%)	
History of abdominal surgery (*n*)	5 (7.9%)	

**Table 2:** Surgical procedures.

	*n* (%)
Nephrectomy	24 (38.1)
Donor nephrectomy	4 (6.3)
Nephroureterectomy	9 (14.3)
Nephroureterectomy + cystectomy	2 (3.2)
Partial nephrectomy	5 (7.9)
Adrenalectomy	10 (15.9)
Cystectomy	5 (7.9)
Prostatectomy	2 (3.2)
Pyeloplasty	1 (1.6)
Renal cyst decortication	1 (1.6)

**Table 3:** Analyses of laboratory data.

	Before surgery mean ± SD (range)	Day 7 mean ± SD (range)	*P* value
PT (%) (88–123)^a^	115 ± 12 (91–155)	103 ± 10 (82–136)	<0.0001
APTT (%) (62–148)^a^	98.8 ± 15 (57–142)	90.9 ± 18 (52–143)	0.0108
D-dimer (*μ*g/mL) (<0.5)^a^	0.7 ± 0.7 (0.01–3.65)	4.9 ± 3.6 (1.32–14.3)	<0.0001

^a^Normal range.
